# ^44^Sc-PSMA-617 for radiotheragnostics in tandem with ^177^Lu-PSMA-617—preclinical investigations in comparison with ^68^Ga-PSMA-11 and ^68^Ga-PSMA-617

**DOI:** 10.1186/s13550-017-0257-4

**Published:** 2017-01-19

**Authors:** Christoph A. Umbricht, Martina Benešová, Raffaella M. Schmid, Andreas Türler, Roger Schibli, Nicholas P. van der Meulen, Cristina Müller

**Affiliations:** 1 Center for Radiopharmaceutical Sciences ETH-PSI-USZ, Paul Scherrer Institut, 5232 Villigen-PSI, Switzerland; 2 Department of Chemistry and Applied Biosciences, ETH Zurich, Zurich, Switzerland; 3 Laboratory of Radiochemistry, Paul Scherrer Institut, Villigen-PSI, Switzerland; 4 Department of Chemistry and Biochemistry University of Bern, Bern, Switzerland

**Keywords:** ^44^Sc, ^68^Ga, ^177^Lu, Prostate cancer, PSMA, PET imaging, Theragnostics, Cyclotron

## Abstract

**Background:**

The targeting of the prostate-specific membrane antigen (PSMA) is of particular interest for radiotheragnostic purposes of prostate cancer. Radiolabeled PSMA-617, a 1,4,7,10-tetraazacyclododecane-*N*,*N′*,*N′′*,*N′′′*-tetraacetic acid (DOTA)-functionalized PSMA ligand, revealed favorable kinetics with high tumor uptake, enabling its successful application for PET imaging (^68^Ga) and radionuclide therapy (^177^Lu) in the clinics. In this study, PSMA-617 was labeled with cyclotron-produced ^44^Sc (*T*_1/2_ = 4.04 h) and investigated preclinically for its use as a diagnostic match to ^177^Lu-PSMA-617.

**Results:**

^44^Sc was produced at the research cyclotron at PSI by irradiation of enriched ^44^Ca targets, followed by chromatographic separation. ^44^Sc-PSMA-617 was prepared under standard labeling conditions at elevated temperature resulting in a radiochemical purity of >97% at a specific activity of up to 10 MBq/nmol. ^44^Sc-PSMA-617 was evaluated in vitro and compared to the ^177^Lu- and ^68^Ga-labeled match, as well as ^68^Ga-PSMA-11 using PSMA-positive PC-3 PIP and PSMA-negative PC-3 flu prostate cancer cells. In these experiments it revealed similar in vitro properties to that of ^177^Lu- and ^68^Ga-labeled PSMA-617. Moreover, ^44^Sc-PSMA-617 bound specifically to PSMA-expressing PC-3 PIP tumor cells, while unspecific binding to PC-3 flu cells was not observed. The radioligands were investigated with regard to their in vivo properties in PC-3 PIP/flu tumor-bearing mice. ^44^Sc-PSMA-617 showed high tumor uptake and a fast renal excretion. The overall tissue distribution of ^44^Sc-PSMA-617 resembled that of ^177^Lu-PSMA-617 most closely, while the ^68^Ga-labeled ligands, in particular ^68^Ga-PSMA-11, showed different distribution kinetics. ^44^Sc-PSMA-617 enabled distinct visualization of PC-3 PIP tumor xenografts shortly after injection, with increasing tumor-to-background contrast over time while unspecific uptake in the PC-3 flu tumors was not observed.

**Conclusions:**

The in vitro characteristics and in vivo kinetics of ^44^Sc-PSMA-617 were more similar to ^177^Lu-PSMA-617 than to ^68^Ga-PSMA-617 and 68Ga-PSMA-11. Due to the almost four-fold longer half-life of ^44^Sc as compared to ^68^Ga, a centralized production of ^44^Sc-PSMA-617 and transport to satellite PET centers would be feasible. These features make ^44^Sc-PSMA-617 particularly appealing for clinical application.

**Electronic supplementary material:**

The online version of this article (doi:10.1186/s13550-017-0257-4) contains supplementary material, which is available to authorized users.

## Background

**Fig. 1:**
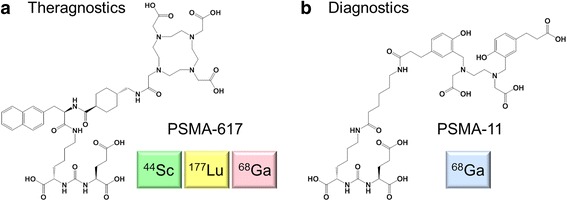
Chemical structures of **a** PSMA-617 and **b** PSMA-11 suitable for labeling with the corresponding radionuclides for theragnostic or diagnostic intervention

Prostate cancer is the second most frequently diagnosed cancer type in men in the US [1]. Patients with localized disease have a good prognosis for survival; however, those diagnosed with metastatic castration-resistant prostate cancer, have much less chance of successful treatment [1, 2]. Sensitive and specific imaging tools are needed in order to enable tumor localization and staging of the disease, as well as monitoring therapy [3]. Currently, nuclear imaging of prostate cancer and related metastases is performed using ^11^C- and ^18^F-choline for positron emission tomography (PET) and ^99m^Tc-methanediphosphonic acid for single photon emission computed tomography (SPECT), respectively [4, 5]. However, the clinical value of these radiotracers has been controversially discussed due to the low specificity and sensitivity [6, 7]. More promising may be a class of radioligands for targeting prostate-specific membrane antigen (PSMA) that has been widely investigated over the last two decades [8–10]. PSMA is a transmembrane protein, upregulated in poorly differentiated, metastatic, and hormone-refractory prostate carcinomas, while physiological expression is restricted to only a few sites, including the kidneys [11]. In recent years, a number of PSMA-targeted nuclear imaging agents were developed [12–14], among those PSMA-11, which comprises an acyclic *N*,*N′*-bis-[2-hydroxy-5-(carboxyethyl)benzyl]ethylenediamine-*N*,*N′*-diacetic acid (HBED-CC)-chelator suitable for coordination of ^68^Ga (*T*_1/2_ = 68 min, Eβ^+^_av_ = 830 keV; Fig. 1, Table 1) [15, 16]. This PSMA radioligand has been used successfully in clinics for PET imaging of prostate cancer [17–19]. More recently, a structurally modified PSMA ligand, referred to as PSMA-617, has been designed with a 1,4,7,10-tetraazacyclododecane-*N*,*N′*,*N′′*,*N′′′*-tetraacetic acid (DOTA) chelator (Fig.1, Table 1). This derivative allows coordination of diagnostic and therapeutic radionuclides and, hence, it paved the way towards a theragnostic approach [20, 21]. Clinical studies performed so far demonstrated the promising potential of ^68^Ga- and ^177^Lu-labeled PSMA-617 to be used for the management of prostate cancer (Table 1) [21, 22]. ^68^Ga-PSMA-11 and ^68^Ga-PSMA-617 showed similar accumulation in most organs, albeit the renal clearance of ^68^Ga-PSMA-617 was significantly faster than for ^68^Ga-PSMA-11 [20, 21]. In patients, application of ^68^Ga-PSMA-11 resulted in an increased contrast on delayed images; however, all lesions were already visible on the 1 h post injection (p.i.) scans [21, 23]. On the other hand, it was suggested to scan patients 2–3 h after injection of ^68^Ga-PSMA-617 due to the improving image quality over time [21]. For the same reason, the scanning at even later time points (>3 h p.i.) was supposed to enable discovering additional lesions [21]; however, longer-lived radionuclides would be required for this purpose. Several ^18^F-based PSMA-ligands are currently under development, and the first clinical applications with ^18^F-DCFPyl and ^18^F-PSMA-1007 revealed promising results [24, 25]. The concept of radiotheragnostics, as proposed herein, is based on the use of a diagnostic and a therapeutic radionuclide with the same PSMA-targeting ligand.

**Table 1:** Overview of radionuclides and corresponding ligands used in this study

Nuclide	Half-life	Radiation energy	Ligand	Chelator	Application	Refs.
^44^Sc	4.04 h	Eβ^+^_av_ = 632 keV Eγ = 1157 keV*	PSMA-617	DOTA	PET (β^+^-radiation)	Evaluated in this study
^177^Lu	6.65 days	Eβ^−^_av_ = 134 keV Eγ = 113 keV, 208 keV	PSMA-617	DOTA	Therapy/SPECT (β^−^/γ-radiation)	[22, 37–39]
^68^Ga	68 min	Eβ^+^_av_ = 830 keV Eγ = 1077 keV**	PSMA-617 PSMA-11	DOTA HBED-CC	PET (β^+^-radiation)	[21] [17, 18, 40]

*I = 99.9%; **I = 3.2%

In this regard, we proposed ^44^Sc (*T*_1/2_ = 4.04 h, Table 1 [26]) as an alternative radionuclide to ^68^Ga for PET imaging allowing to use ^44^Sc-PSMA-617 as a diagnostic match to ^177^Lu-PSMA-617. The emitted positrons have a lower energy (Eβ^+^_av_ = 632 keV) as compared to ^68^Ga (Eβ^+^_av_ = 830 keV), enabling PET imaging with a potentially favorable spatial resolution [27, 28]. Recently, the production of ^44^Sc via the ^44^Ca(p,n)^44^Sc nuclear reaction has been implemented at the research cyclotron at Paul Scherrer Institut, providing this radionuclide with high radionuclidic purity (>99%) and at high activities (>2 GBq) [29]. There is great potential of using ^44^Sc clinically, as it can be produced at medical cyclotrons typically installed in PET centers worldwide. Another means of producing ^44^Sc is via the ^44^Ti/^44^Sc generator [30]; however, the quantity of eluted ^44^Sc activity is very limited (<200 MBq) in this case. In contrast, the proposed production route of ^44^Sc at a cyclotron would address the clinical needs and, thus, be a favorable option for application of this radionuclide in patients.

The aim of this study was to investigate PSMA-617, labeled with cyclotron-produced ^44^Sc, in preclinical experiments. ^44^Sc-PSMA-617 was prepared and evaluated for direct comparison with ^177^Lu- and ^68^Ga-PSMA-617, as well as with ^68^Ga-PSMA-11. PSMA-positive (PC-3 PIP) and PSMA-negative (PC-3 flu) prostate cancer cells were used for the performance of in vitro studies and, as xenografts in mice, for preclinical in vivo experiments including PET imaging.

## Methods

### PSMA-ligands and radionuclides

The PSMA ligands PSMA-617 and PSMA-11 were obtained from Advanced Biochemical Compounds (ABX GmbH, Radeberg, Germany). ^44^Sc was prepared by proton irradiation of enriched ^44^Ca targets at the Injector 2 cyclotron at PSI, as previously reported [29]. The irradiation of targets with ~11 MeV protons at a beam current of 50 μA lasted for 90 min. The separation of the produced ^44^Sc from the target material was carried out by chromatographic methods using DGA resin [29]. ^44^Sc was provided in an acidic solution (~0.1 M HCl, pH ~1 in ~700 μL) and was used directly for labeling reactions. No-carrier added ^177^Lu in HCl 0.5 M was provided by Isotope Technologies Garching (ITG GmbH, Germany). ^68^Ga was obtained from a ^68^Ge/^68^Ga generator (Eckert & Ziegler, Berlin, Germany) using 0.1 M HCl as an eluent (radionuclidic purity >99%).

### Radiolabeling of PSMA-617 and PSMA-11

PSMA-617 was labeled with ^44^Sc, ^68^Ga, and ^177^Lu in a mixture of sodium acetate (0.5 M, pH 8) and HCl (0.05–0.1 M, pH ~1) at a pH of 3.5–4.5. The reaction mixture was incubated for 10 min at 95 °C. PSMA-11 was labeled with ^68^Ga under the same reaction conditions. Quality control of the radiolabeled PSMA ligands was performed using high-performance liquid chromatography (HPLC) with a C-18 reversed-phase column (Xterra^TM^ MS, C18, 5 μm, 150 × 4.6 mm; waters) (Additional file 1).

### Determination of n-octanol/PBS distribution coefficients

The distribution coefficients (logD values) of the radioligands were determined by a shake-flask method using liquid-liquid extraction followed by phase separation, as previously reported (Additional file 1) [31]. In brief, the PSMA ligands were labeled at a specific activity of 5 MBq/nmol. Samples containing 1.25 MBq (250 pmol) of the radioligand in a volume of 25 μL were added to each vial containing 1475 μL of PBS (pH 7.4) and 1500 μL of n-octanol. The vials were vortexed vigorously for 1 min and then centrifuged for 6 min for phase separation. The concentration of radioactivity in a defined volume of each layer was measured in a γ-counter (Perkin Elmer, Wallac Wizard 1480). The distribution coefficients were expressed as the logarithm of the ratio of counts per minute (cpm) measured in the n-octanol phase to the cpm measure in the PBS phase. The values are reported as the average of at least three independent measurements (± standard deviation, SD), each performed with five replicates. Data were analyzed for significance using a one-way ANOVA test (GraphPad Prism software, version 7). A *p* value of <0.05 was considered as statistically significant.

### Cell culture

The PC-3 PIP (PSMA^pos^) and PC-3 flu (PSMA^neg^) tumor cells were kindly provided by Prof. Dr. Martin Pomper (John Hopkins Institutions, Baltimore, USA) [32]. The cells were grown in RPMI cell culture medium supplemented with 10% fetal calf serum, L-glutamine, antibiotics, and puromycin (2 μg/mL) to maintain PSMA expression (Additional file 1) [33].

### Cell experiments

Determination of the PSMA affinity was performed by saturation binding assays using PC-3 PIP cells and different concentrations of ^nat/44^Sc-, ^nat/177^Lu-, ^nat/68^Ga-PSMA-617, or ^nat/68^Ga-PSMA-11, respectively (Additional file 1). The relative affinities were defined as the average of at least three independent experiments and expressed as inverse molar ratio of compound needed for half-maximal binding to PSMA and the relative affinity of ^177^Lu-PSMA-617 was set to 1.

Cell uptake and internalization experiments were performed with ^44^Sc-, ^177^Lu-, and ^68^Ga-PSMA-617 as well as ^68^Ga-PSMA-11 using PSMA^pos^ PC-3 PIP and PSMA^neg^ PC-3 flu cells in order to investigate whether they behaved equally and whether the uptake was PSMA-specific. For this purpose, cells were seeded in 12-well plates (~5 × 10^5^ cells in 2 mL RPMI medium/well) allowing adhesion and growth overnight at 37 °C. After removal of the supernatant, cells were washed once with PBS prior to the addition of RPMI medium without supplements (975 μL/well), followed by the addition of the radiolabeled PSMA ligands (MBq/nmol) to each well (25 μL, 7.5 pmol). Some of the cell samples were co-incubated with excess 2-(phosphonomethyl)pentane-1,5-dioic acid (2-PMPA; 100 μM) to block PSMA on the surface of PC-3 PIP cells. The well plates were incubated at 37 °C for 2 and 4 h, respectively. The cells were washed three times with ice-cold PBS to determine the total uptake of the radioligands (PSMA-bound fraction on the surface and internalized fraction). The internalized fraction of the radioligands was determined in cells which were washed with ice-cold PBS, then incubated for 10 min with acidic stripping buffer (0.05 M glycine stripping buffer in 100 mM NaCl, pH 2.8) followed by an additional washing step with ice-cold PBS. Cell samples were lysed by addition of NaOH (1 M, 1 mL) to each well. The samples of the cell suspensions were measured in a γ-counter (Perkin Elmer, Wallac Wizard 1480). After homogenization of the cell suspensions, the protein concentration was determined for each sample using a Micro BCA Protein Assay kit (Pierce, Therma Scientific). The results were expressed as percentage of total added radioactivity per 300 μg/mL protein.

### Tumor mouse model

In vivo experiments were approved by the local veterinarian department and conducted in accordance with the Swiss law of animal protection. All mice were obtained from Charles River Laboratories (Sulzfeld, Germany), at the age of 5–6 weeks. Female, athymic nude Balb/c mice were subcutaneously inoculated with PC-3 PIP cells (6 × 10^6^ cells in 100 μL Hank’s balanced salt solution (HBSS) with Ca^2+^/Mg^2+^) on the right shoulder and with PC-3 flu cells (5 × 10^6^ cells in 100 μL HBSS with Ca^2+^/Mg^2+^) on the left shoulder 2 weeks before the performance of the experiments.

### Biodistribution studies

^44^Sc-, ^177^Lu-, and ^68^Ga-PSMA-617 as well as ^68^Ga-PSMA-11 were intravenously injected (5 MBq, 1 nmol, 100–200 μL). Mice were sacrificed at different time points post injection (p.i.) of the radioligands. Selected tissues and organs were collected, weighed and measured using a γ-counter. The results were decay-corrected and listed as a percentage of the injected activity per gram of tissue mass (% IA/g).

### Imaging studies

PET/CT scans were performed using a small-animal bench-top PET/CT scanner (G8, Sofie Biosciences, Culver City, California, USA, and Perkin Elmer, Massachusetts, USA) and a small-animal SPECT/CT scanner (NanoSPECT/CT^TM^, Mediso Medical Imaging Systems, Budapest, Hungary), respectively (Additional file 1). During the scans, the mice were anesthetized with a mixture of isoflurane and oxygen.

Static whole-body PET scans of 20 min duration were performed at 30 min, 2 and 4 h after injection of ^44^Sc-PSMA-617 (~5 MBq, 1 nmol) and of 10 min duration at 30 min and 2 h after injection of ^68^Ga-PSMA-617 and ^68^Ga-PSMA-11 (~5 MBq, 1 nmol), respectively. The PET scans were followed by a CT of 1.5 min. The SPECT scan of 45 min duration was performed 2 h after injection of ^177^Lu-PSMA-617 (~50 MBq, 1 nmol) followed by a CT of 7.5 min. Reconstruction of the acquired data was performed by using the software of the respective scanner.

All images were prepared using *VivoQuant* post-processing software (version 2.10, inviCRO Imaging Services and Software, Boston USA). A Gauss post-reconstruction filter (full width at half maximum = 1 mm) was applied to the images, which were presented with the scale adjusted to allow visualization of the most important organs and tissues, usually by cutting 0.5–1% of the lower scale.

## Results

### Radiolabeling and in vitro evaluation of PSMA-targeted radioligands

The radiochemical yield was always >97% for all radiolabeled compounds at a specific activity of up to 10 MBq/nmol (Additional file 1: Figure S1). The n-octanol/PBS distribution coefficients (logD values) were in the same range for ^44^Sc-, ^177^Lu-, and ^68^Ga-PSMA-617, but somewhat reduced for ^68^Ga-PSMA-11 (Table 2). The K_D_ values obtained for ^44^Sc-PSMA-617 and ^177^Lu-PSMA-617 were in the same range, but somewhat higher values were determined for the ^68^Ga-labeled PSMA ligands (Additional file 1: Figure S2). The results were converted into relative PSMA-binding affinities, which were similar for ^44^Sc-PSMA-617 and ^177^Lu-PSMA-617, but slightly reduced for the ^68^Ga-labeled PSMA ligands (Table 2).

**Table 2:** In vitro characteristics of the radiolabeled PSMA-ligands

Radioligand	LogD values^a^	Relative PSMA-binding affinity^b^
^44^Sc-PSMA-617	−4.21 ± 0.04	1.2
^177^Lu-PSMA-617	−4.18 ± 0.06	1.0
^68^Ga-PSMA-617	−4.30 ± 0.10	0.5
^68^Ga-PSMA-11	−4.82 ± 0.07	0.4

^a^LogD values represent the average (±SD) of 3–5 independent experiments performed in triplicate

^b^Binding affinity is the inverse molar ratio of the average K_D_ values determined in four independent experiments performed in triplicates

### Cell internalization studies of PSMA radioligands

**Fig. 2:**
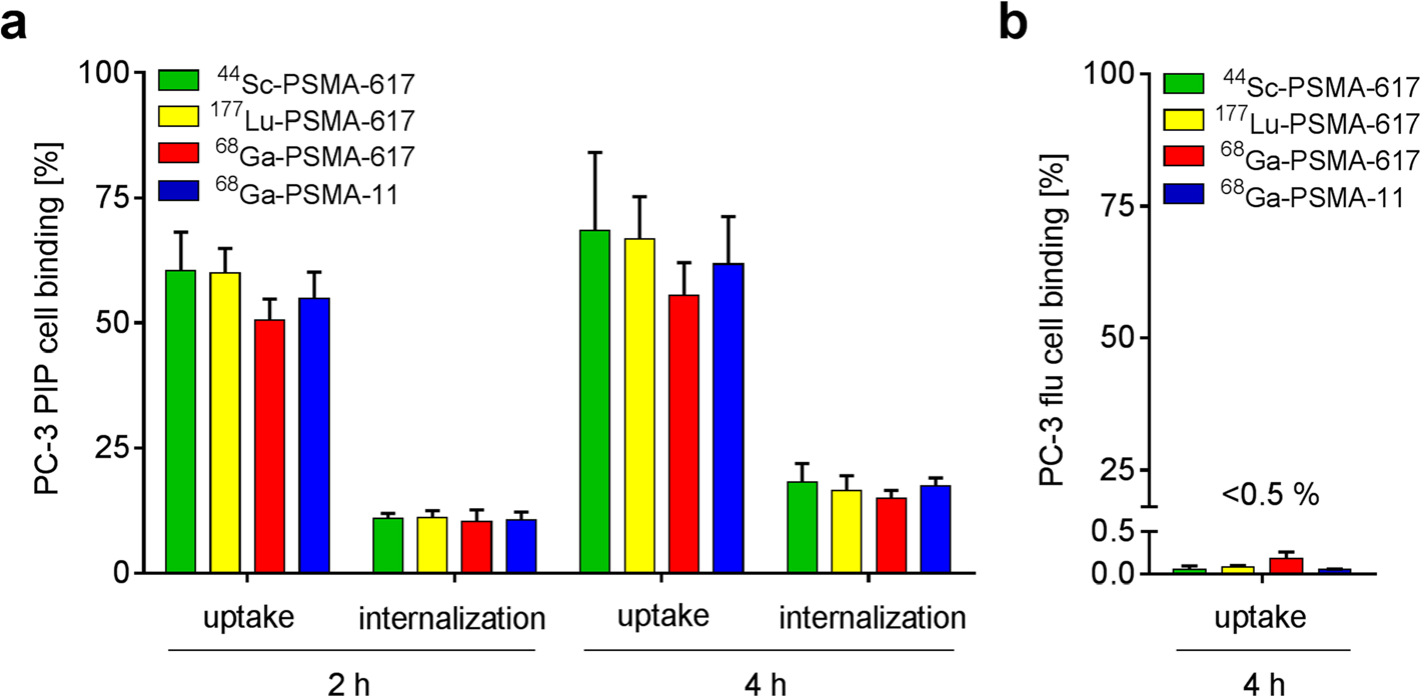
Uptake and internalization of all radioligands using **a** PSMA^pos^ PC-3 PIP and **b** PSMA^neg^ PC-3 flu tumor cells

Uptake and internalization of all four radioligands was investigated using PC-3 PIP/flu cells (Fig. 2). The uptake of all radioligands into PC-3 PIP cells (PSMA^pos^) was comparable and in the range of 55–70%, whereas the internalized fraction was about 10–15% of total added activity (Fig. 2a). The uptake of all radioligands dropped to <0.5% when PC-3 flu cells (PSMA^neg^) were used, which proves PSMA-specific uptake/internalization of all four radioligands (Fig. 2b).

### Biodistribution studies in tumor-bearing mice

**Fig. 3:**
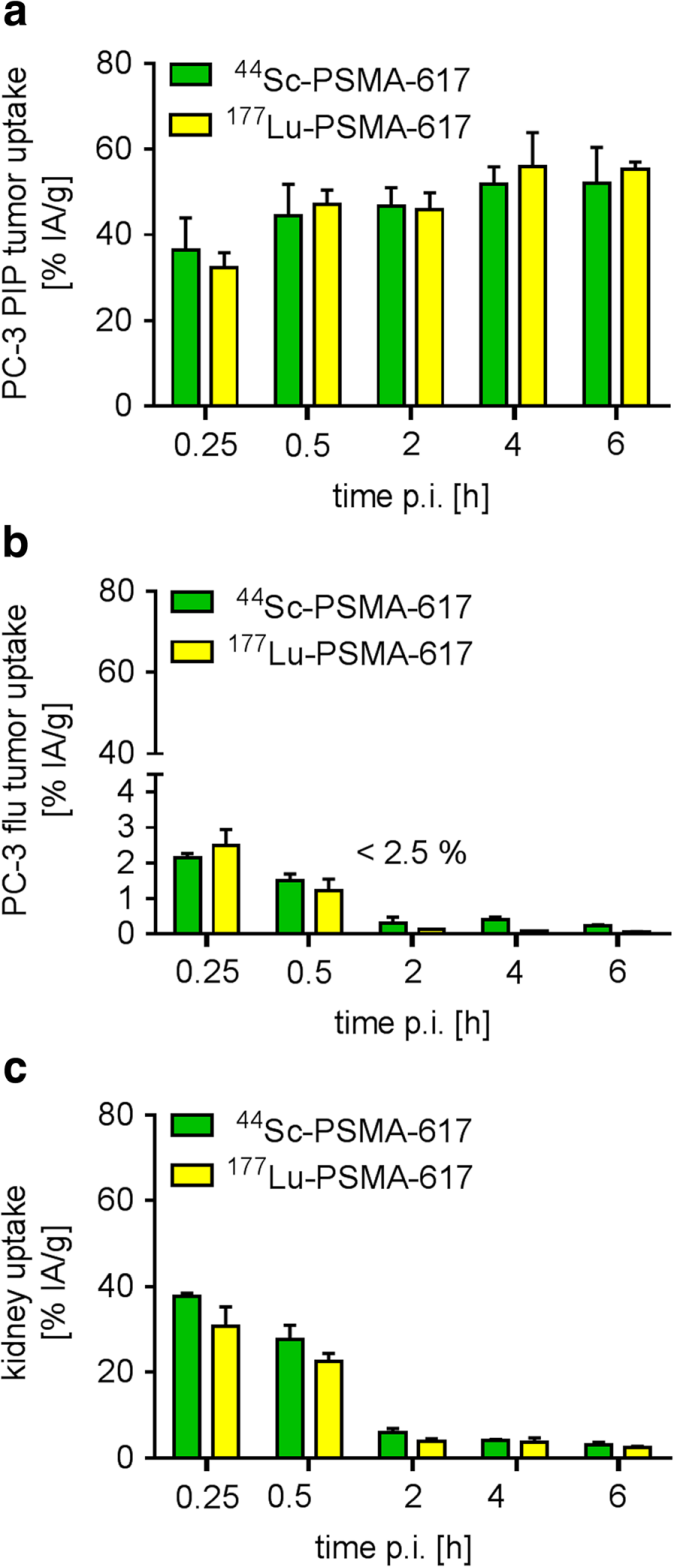
Biodistribution data of ^44^Sc-PSMA-617 and ^177^Lu-PSMA-617, respectively, in PC-3 PIP/flu tumor-bearing mice. **a** Uptake of the radioligands in PSMA^pos^ PC-3 PIP tumors, **b** in PSMA^neg^ PC-3 flu tumors, and **c** clearance through the kidneys

The tissue distribution profiles of ^44^Sc-PSMA-617 and ^177^Lu-PSMA-617 were investigated in PC-3 PIP/flu tumor-bearing mice over a period of 6 h (Fig. 3, Additional file 1: Tables S1/S2). Relatively high radioactivity levels were detected in the blood shortly after injection (~7% IA/g, 15 min p.i.); however, blood activity decreased quickly over time to less than 0.5% IA/g at 2 h p.i. The uptake of both radioligands in PC-3 PIP tumors was already high 15 min after injection (36.5 ± 7.44 and 32.3 ± 3.54% IA/g) and increased further to reach a maximum uptake (51.9 ± 4.05 and 56.0 ± 8.0% IA/g) after 4 h (Fig. 3a). In PC-3 flu tumor xenografts, however, the accumulated activity was clearly below the blood level, indicating that unspecific accumulation of the radioligands did not occur (Fig. 3b). The uptake of the radioligands in the kidneys (37.7 ± 0.82 and 30.8 ± 4.52% IA/g, 15 min p.i.) was cleared quickly, resulting in renal retention of ~6% IA/g after 2 h and ~3% IA/g after 6 h (Fig. 3c). Most importantly, the tissue distribution kinetics and excretion pattern of ^44^Sc-PSMA-617 was comparable to that of ^177^Lu-PSMA-617 (Fig. 3).

**Fig. 4:**
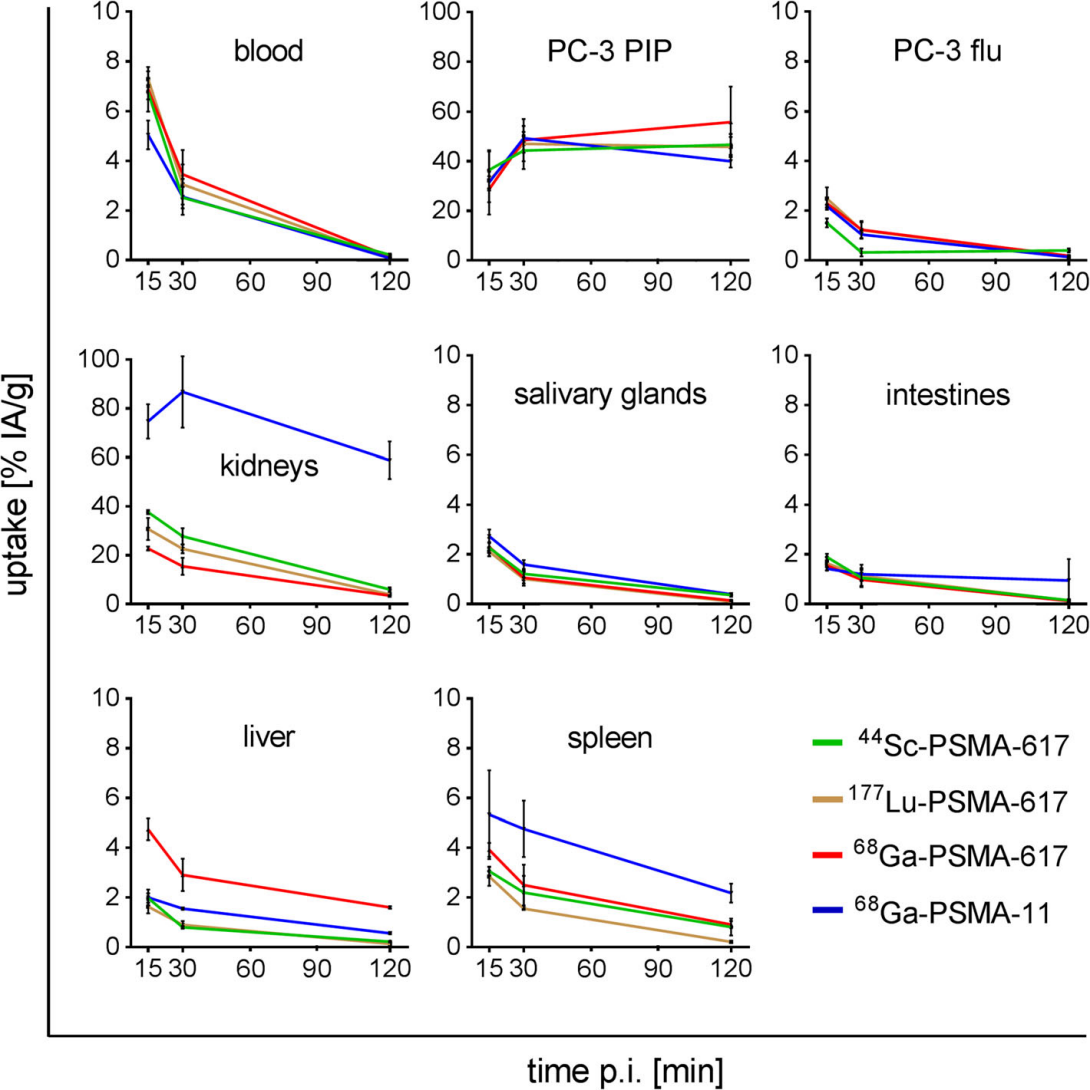
Time-dependent uptake and retention of radioactivity in different organs and tissues over the first 2 h after injection of ^44^Sc-PSMA-617 (*green*), ^177^Lu-PSMA-617 (*yellow-brown*), ^68^Ga-PSMA-617 (*red*) and ^68^Ga-PSMA-11 (*blue*)

All four radioligands were investigated under the same in vivo conditions over a period of 2 h (Fig. 4, Table 3, Additional file 1: Tables S1–S5). Clearance of radioactivity from the blood pool was fast (<0.5% IA/g, 2 h p.i) resulting in very high tumor-to-blood ratios (>200) at 2 h p.i. The accumulation of activity in PC-3 PIP tumors was comparable for all radioligands (45–49% IA/g, 2 h p.i.). The tumor-to-background ratios of ^44^Sc-PSMA-617 were virtually the same as those of ^177^Lu-PSMA-617. Renal retention of ^44^Sc-PSMA-617 was comparable to the ^68^Ga- and ^177^Lu-labeled versions; however, ^68^Ga-PSMA-11 showed a much higher uptake in the kidneys (~60% IA/g, 2 h p.i., Fig. 4). This resulted in reduced tumor-to-kidney ratios of ^68^Ga-PSMA-11 as compared to the other radioligands. Moreover, uptake of ^68^Ga-PSMA-11 in the spleen was also slightly increased. In comparison to ^44^Sc-PSMA-617 and ^177^Lu-PSMA-617, ^68^Ga-PSMA-617 showed reduced tumor-to-liver ratios which is due to the increased liver uptake of this radioligand.

**Table 3:** Tumor-to-background ratios obtained in PC-3 PIP/flu tumor-bearing mice

	15 min after injection			
PSMA-ligand	^44^Sc-PSMA-617	^177^Lu-PSMA-617	^68^Ga-PSMA-617	^68^Ga-PSMA-11
Tumor-to-blood	5.38 ± 0.90	4.45 ± 0.66	4.15 ± 0.24	6.10 ± 1.84
Tumor-to-liver	18.8 ± 5.02	20.4 ± 5.48	6.02 ± 0.59	15.3 ± 3.96
Tumor-to-kidney	0.97 ± 0.21	1.06 ± 0.14	1.26 ± 0.25	0.41 ± 0.14
	30 min after injection			
	^44^Sc-PSMA-617	^177^Lu-PSMA-617	^68^Ga-PSMA-617	^68^Ga-PSMA-11
Tumor-to-blood	17.6 ± 1.23	16.0 ± 3.52	16.9 ± 4.22	20.5 ± 6.40
Tumor-to-liver	55.6 ± 7.36	53.1 ± 6.68	19.0 ± 2.32	31.7 ± 2.48
Tumor-to-kidney	1.60 ± 0.22	2.09 ± 0.24	3.62 ± 0.81	0.58 ± 0.10
	2 h after injection			
	^44^Sc-PSMA-617	^177^Lu-PSMA-617	^68^Ga-PSMA-617	^68^Ga-PSMA-11
Tumor-to-blood	>200	>200	>200	>200
Tumor-to-liver	>200	>200	35.2 ± 9.94	71.3 ± 7.48
Tumor-to-kidney	7.98 ± 1.71	11.6 ± 0.87	15.9 ± 3.26	0.69 ± 0.12

### Imaging studies in PC-3 PIP/flu tumor-bearing mice

**Fig. 5:**
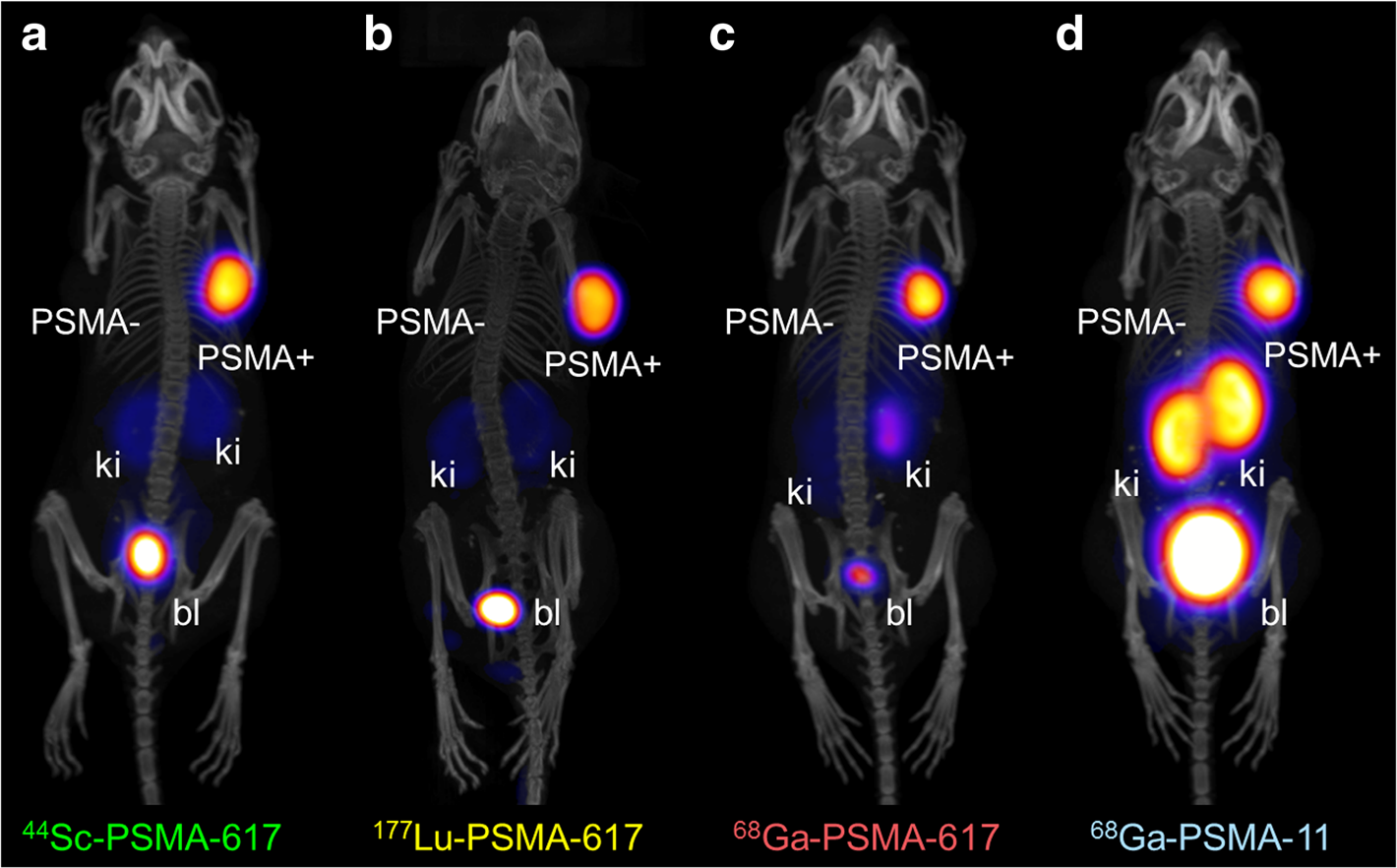
Images (PET/CT and SPECT/CT, respectively) as maximum intensity projections (MIPs) of PC-3 PIP/flu tumor-bearing mice 2 h after injection of **a** ^44^Sc-PSMA-617, **b** ^177^Lu-PSMA-617, **c** ^68^Ga-PSMA-617, and **d** ^68^Ga-PSMA-11. *PSMA*- PC-3 flu tumor, *PSMA+* PC-3 PIP tumor, *ki* kidney, *bl* urinar bladder

Nuclear imaging studies were performed with PC-3 PIP/flu tumor-bearing mice 2 h after injection of ^44^Sc-, ^177^Lu-, and ^68^Ga-PSMA-617 as well as ^68^Ga-PSMA-11 (Fig. 5, Additional file 1: Figure S3). PC-3 PIP tumor xenografts, located on the right shoulder, showed high uptake of activity in all four cases. No activity was detected, however, in PC-3 flu tumors on the left shoulder, demonstrating the PSMA-specific tumor accumulation of the radioligands. The PET images obtained with ^44^Sc- and ^68^Ga-PSMA-617 as well as the SPECT image obtained with ^177^Lu-PSMA-617 showed a similar distribution pattern and confirmed the post mortem data. The tissue distribution of ^68^Ga-PSMA-11 was, however, different in that it accumulated to a significantly higher extent in the kidneys.

**Fig. 6:**
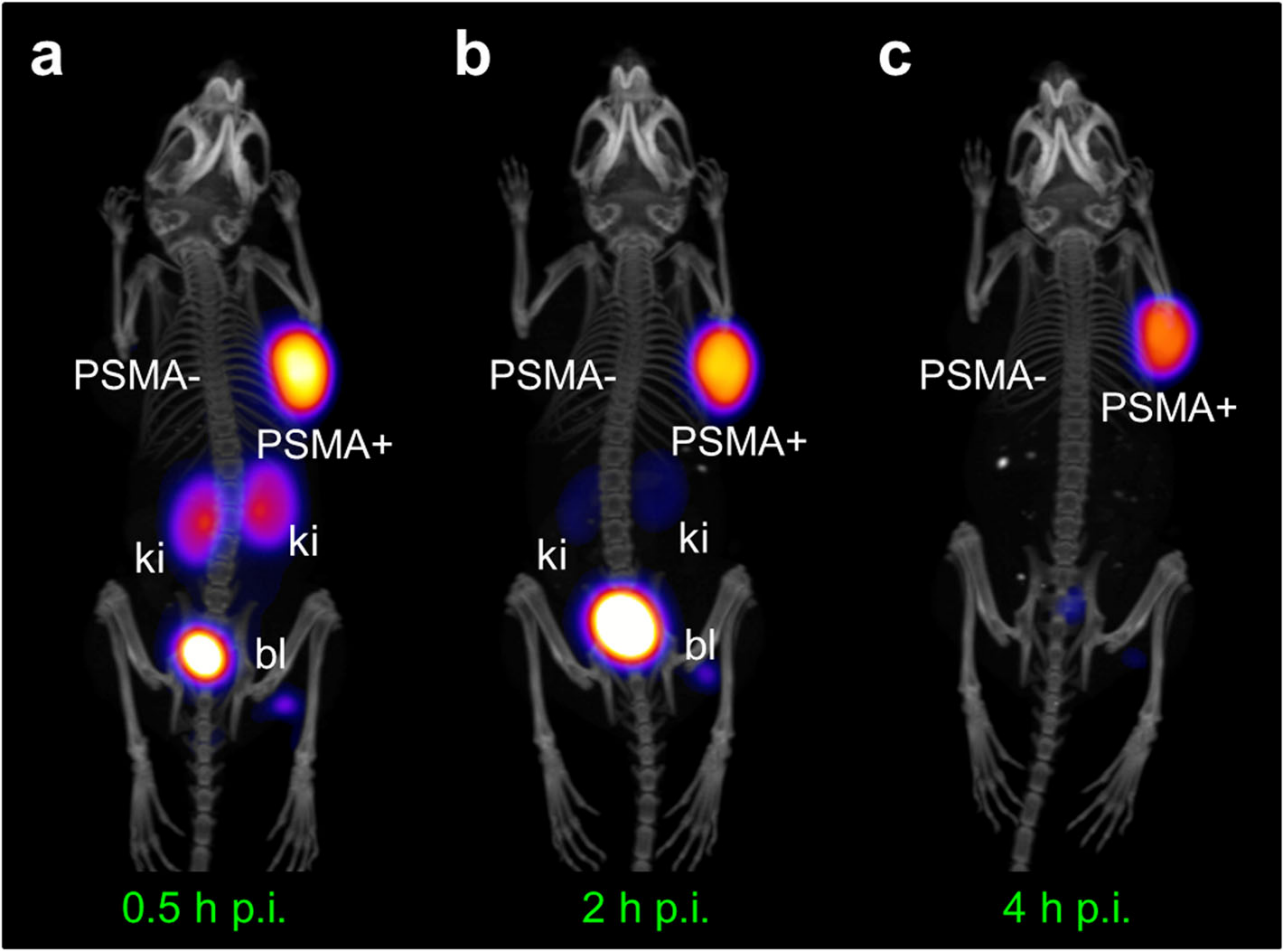
PET/CT images as maximum intensity projections (MIPs) of PC-3 PIP/flu tumor-bearing mice **a** 0.5, **b** 2, and **c** 4 h after injection of ^44^Sc-PSMA-617. *PSMA-* PC-3 flu tumor, *PSMA+* PC-3 PIP tumor, *ki* kidney, *bl* urinary bladder

PET/CT scans were performed at 30 min, 2 and 4 h after injection of ^44^Sc-PSMA-617 to visualize the tissue distribution in the same animal over time (Fig. 6). The PC-3 PIP tumor (PSMA^pos^) was visible already 30 min p.i.; however, at this time point, retention of radioactivity was also seen in the kidneys. At delayed time points, when renal activity was cleared, the tumor became better visible and was finally the only site showing accumulated activity. These results demonstrated increasing tumor-to-background contrast over time and confirmed the advantage of late imaging.

## Discussion

In this study, ^44^Sc-PSMA-617 was compared to ^68^Ga-PSMA-617 and ^68^Ga-PSMA-11 in vitro and in tumor-bearing mice as a potential diagnostic match to the therapeutically employed ^177^Lu-PSMA-617 (Fig. 1). The PC-3 PIP/flu tumor cells enabled the investigation of PSMA-specific and -unspecific uptake of the radioligands in vitro and in vivo by using a PC-3 PIP and PC-3 flu tumor xenograft in the same animal.

A comparable in vitro behavior of ^44^Sc-PSMA-617 and ^177^Lu-PSMA-617, as was determined in this study, was expected due to the chemical similarities of ^44^Sc and ^177^Lu with regard to their complexation using DOTA [34]. Our data revealed almost identical characteristics of ^44^Sc-PSMA-617 and ^177^Lu-PSMA-617 with regard to their hydrophilic properties and PSMA-binding affinities (Table 2). ^44^Sc-PSMA-617 showed PSMA-specific cell uptake and internalization, as was the case for all three other radioligands (Fig. 2).

The evaluation of the radioligands in mice revealed a favorable tumor accumulation of ^44^Sc-PSMA-617 already shortly after injection and a relatively fast clearance of background activity through the kidneys (Fig. 4). These circumstances enabled PET/CT imaging with increasing tumor-to-background contrast over time (Fig. 6). The tissue distribution profile of ^44^Sc-PSMA-617 was largely identical to that obtained with ^177^Lu-PSMA-617 over the investigated period of 6 h (Fig. 3). Comparable pharmacokinetic properties of ^44^Sc- and ^177^Lu-labeled compounds were expected as previously shown in preclinical experiments with a DOTA-functionalized folate conjugate [35]. The chemical similarities of these nuclides and, as a consequence, comparable pharmacokinetics of ligands labeled with ^44^Sc and ^177^Lu, respectively, would thus, allow predicting the exact tissue distribution of ^177^Lu-PSMA-617 based on the PET imaging results obtained with ^44^Sc-PSMA-617.

The chemical structure of ^68^Ga-PSMA-11 is fundamentally different (Fig. 1). Not surprisingly, the tissue distribution profile of ^68^Ga-PSMA-11 in mice varied significantly from those of the other investigated radioligands, as was observed previously in preclinical experiments [15, 20]. High uptake of activity was found in the kidneys promptly after injection of ^68^Ga-PSMA-11 (Fig. 4). This observation was in agreement with the fact that in patients ^68^Ga-PSMA-11 showed an increased retention of activity in the kidneys as compared to ^68^Ga-PSMA-617 [21, 23].

Even when using the same chelator, the chemical properties of ^68^Ga- and ^177^Lu-labeled compounds are not identical due to the different coordination chemistry of these radiometals [34] which may potentially result in different in vivo kinetics [36]. In the case of ^68^Ga-PSMA-617, the pharmacokinetics were similar to those of the ^44^Sc- and ^177^Lu-labeled versions, with the exception of an increased uptake of ^68^Ga-PSMA-617 in the liver (Fig. 4). Exactly the same phenomenon has been previously observed in mice, when they were injected with ^68^Ga-labeled DOTA peptides [28]. In patients, increased hepatic uptake of radioactivity was not detected after injection of ^68^Ga-PSMA-617 [21, 23].

Although ^68^Ga-labeled PSMA ligands—in particular ^68^Ga-PSMA-11—are successfully employed in the clinics, it is indisputable that the high cost for a ^68^Ge/^68^Ga generator is a disadvantage when considering the limited activity that can be eluted daily. The short half-life of ^68^Ga is also a limiting factor, making transportation of activity over long distances unattractive. The almost fourfold longer half-life of ^44^Sc as compared to ^68^Ga would enable the shipment of ^44^Sc-based radiopharmaceuticals to PET centers without cyclotron and radiopharmaceutical facilities. Concerns of radiation safety with regard to the high-energy γ-radiation of ^44^Sc (E_γ_ = 1157 keV) may be addressed by using tungsten-based containers as are employed for other commercial PET nuclides such as ^89^Zr, which emits high-energy γ-radiation (E_γ_ = 909 keV) as well.

The use of ^44^Sc-PSMA-617 as a diagnostic radioligand would be novel and favorable, since the longer half-life of ^44^Sc permits a greater flexibility of patient scheduling to accommodate urgent cases in between, thus, allowing optimization of patient management in nuclear medicine departments. Moreover, ^44^Sc-PSMA-617 would allow PET/CT imaging of patients several hours after injection, potentially allowing dosimetry estimations and enabling the detection of small pathological lesions due to increased tumor-to-background ratios and, consequently, improved image contrast.

A clinical translation of the proposed concept, as is planned for the near future, will allow addressing the diverse aspects of this promising approach. Appropriate acquisition parameters for PET imaging of patients with prostate cancer remain to be determined after collecting practical experiences with ^44^Sc-PSMA-617 in a clinical setting.

## Conclusions

In this study, the potential of ^44^Sc-PSMA-617 for PET imaging was demonstrated in a preclinical setting. The results indicate more similar characteristics of ^44^Sc-PSMA-617 to ^177^Lu-PSMA-617 than is the case for ^68^Ga-PSMA-11. An important advantage of using ^44^Sc over ^68^Ga is the feasibility of transporting ^44^Sc-based PSMA-ligands to PET centers without cyclotron and radiopharmaceutical facilities due to its longer half-life. The fact that ^44^Sc-PSMA-617 can enable delayed PET imaging makes it particularly appealing for clinical application as it may allow pretherapeutic dosimetry and an improved image quality at later time points after injection.

## Additional file

Additional file 1: Supporting information. (DOCX 671 kb)

## Abbreviations

2-PMPA: 2-(Phosphonomethyl)pentane-1,5-dioic acid
PSMA: prostate-specific membrane antigen
DOTA: 1,4,7,10-Tetraazacyclododecane- *N*,*N′*,*N′′*,*N′′′*-tetraacetic acid
HBED-CC: *N*,*N′*-bis-[2-hydroxy-5-(carboxyethyl)benzyl]ethylenediamine-*N*,*N′*-diacetic acid
HBSS: Hank’s balanced salt solution
HPLC: High performance liquid chromatography
IA: Injected activity
p.i.: Post injection
PBS: Phosphate-buffered saline pH 7.4
PET: Positron emission tomography
PSMA: Prostate-specific membrane antigen
SPECT: Single photon emission computed tomography
