# Supplementary material for: 44Sc-PSMA-617 for radiotheragnostics in tandem with 177Lu-PSMA-617—preclinical investigations in comparison with 68Ga-PSMA-11 and 68Ga-PSMA-617
Source: EJNMMI Res. 2017 Jan 19;7:9. doi: 10.1186/s13550-017-0257-4 (PMC5247395; doi:10.1186/s13550-017-0257-4)
Supplement: Additional file 1: — Supporting information. (DOCX 671 kb) [file 13550_2017_257_MOESM1_ESM.docx]

**Supporting Information**

**^44^Sc-PSMA-617 for radiotheragnostics in tandem with ^177^Lu-PSMA-617 – preclinical investigations in comparison with ^68^Ga-PSMA-11 and ^68^Ga-PSMA-617**

Christoph A. Umbricht^1^, Martina Benešová^1,2^, Raffaella M. Schmid^1^, Andreas Türler^3,4^, Roger Schibli^1,2^, Nicholas P. van der Meulen^1,3^, Cristina Müller^1,2*^

*^1^Center for Radiopharmaceutical Sciences ETH-PSI-USZ, Paul Scherrer Institut, Villigen-PSI, Switzerland*

*^2^Department of Chemistry and Applied Biosciences, ETH Zurich, Zurich, Switzerland*

*^3^Laboratory of Radiochemistry, Paul Scherrer Institut, Villigen-PSI, Switzerland*

*^4^Department of Chemistry and Biochemistry University of Bern, 3012 Bern, Switzerland*

[christoph.umbricht@psi.ch](mailto:christoph.umbricht@psi.ch); [martina.benesova@psi.ch](mailto:martina.benesova@psi.ch); [raffaella.schmid@psi.ch](mailto:raffaella.schmid@psi.ch);

[andreas.tuerler@psi.ch](mailto:andreas.tuerler@psi.ch); [roger.schibli@psi.ch](mailto:roger.schibli@psi.ch); [nick.vandermeulen@psi.ch](mailto:nick.vandermeulen@psi.ch); [cristina.mueller@psi.ch](mailto:cristina.mueller@psi.ch)

* Corresponding author:

PD Dr. Cristina Müller

Center for Radiopharmaceutical Science ETH/PSI/USZ

Paul Scherrer Institute

5232 Villigen-PSI

Switzerland

e-mail: cristina.mueller@psi.ch

phone: +41-56-310 44 54

fax: +41-56-310 28 49

1. Radiolabeling of PSMA-Targeted Ligands

The radiolabeling of the PSMA ligands (PSMA-617 and PSMA-11) was performed at variable specific activities according to the experiment in which the radioligands were employed.

**Experimental Procedure**

*^44^Sc-labeling of PSMA-617:* A solution of sodium acetate (0.5 M, pH 8) was added to the ^44^Sc eluate (~0.1 M HCl, pH ~1) at a volume ratio of 1:1 to give a pH of 3.5-4.5. PSMA-617 was added from a stock solution (1 mM, in MilliQ water) to obtain a specific activity of 5 MBq/nmol if not otherwise stated (up to 10 MBq/nmol). The reaction mixture was incubated for 10 min at 95 °C. The radiochemical purity was >97% (Fig. S1A).

*^177^Lu-labeling of PSMA-617*: A solution of sodium acetate (0.5 M, pH 8) was added to the ^177^Lu/HCl 0.05 M solution at a volume ratio of 1:5 to obtain a pH of ~4.5. PSMA-617 (1 mM, in MilliQ water) was added to obtain a specific activity of 5 MBq/nmol if not otherwise stated (up to 50 MBq/nmol). The reaction mixture was incubated for 10 min at 95 °C. The radiochemical purity was >98% (Fig. S1B).

*^68^Ga-labeling of PSMA-617*: A solution of sodium acetate (0.5 M, pH 8) was added to the ^68^Ga eluate (~0.1 M HCl, pH ~1) at a volume ratio of 1:2.6 to give a pH of ~4.5. PSMA-617 (1 mM, in MilliQ water) was added to obtain a specific activity of 5 MBq/nmol if not otherwise stated (up to 35 MBq/nmol). The reaction mixture was incubated for 10 min at 95°C. The radiochemical purity was >98% (Fig. S1C).

*^68^Ga-labeling of PSMA-11*: The preparation of ^68^Ga-PSMA-11 was performed in analogy to the preparation of ^68^Ga-PSMA-617. The radiochemical purity was >98% (Fig. S1D).

Quality control of the radiolabeled PSMA ligands was performed using high-performance liquid chromatography (HPLC) with a C-18 reversed-phase column (Xterra^TM^ MS, C18, 5 μm, 150×4.6 mm; Waters). The mobile phase consisted of MilliQ water containing 0.1% trifluoracetic acid (A) and acetonitrile (B) with a gradient of 95% A and 5% B to 20% A and 80% B over a period of 15 min at a flow rate of 1.0 mL/min. The radioligands were diluted in MilliQ water containing sodium diethylenetriamine pentaacetic acid (Na-DTPA; 50 µM) prior to injection into HPLC.

**Results**

The radiolabeling of PSMA-617 with ^44^Sc, ^177^Lu or ^67/68^Ga and of PSMA-11 with ^67/68^Ga was performed according to the required specific activity for a particular experimental setting. HPLC-based quality control was performed after each labeling procedure and the integrated peak area on the chromatograms, representing the radioligand, was always >97%, indicating high radiochemical purity. The retention time of ^44^Sc-PSMA-617 and ^177^Lu-PSMA-617 was identical (t_R_ = 8.7 ± 0.1 min, Fig. S1A/B), while ^68^Ga-PSMA-617 showed a slightly increased retention time (t_R_ = 9.1 ± 0.1 min, Fig. S1C). The retention time of ^68^Ga-PSMA-11 (t_R_ = 7.4 ± 0.1 min) was shorter as compared to the other radioligands which may be explained by the overall more hydrophilic properties of this radioligand (Fig. S1D). The retention time of the diethylenetriamine pentaacetic acid (DTPA)-coordinated radiometals (t_R_ = 2.2 ± 0.1 min) was identical for ^44/47^Sc-DTPA, ^177^Lu-DTPA and ^67/68^Ga-DTPA, respectively.


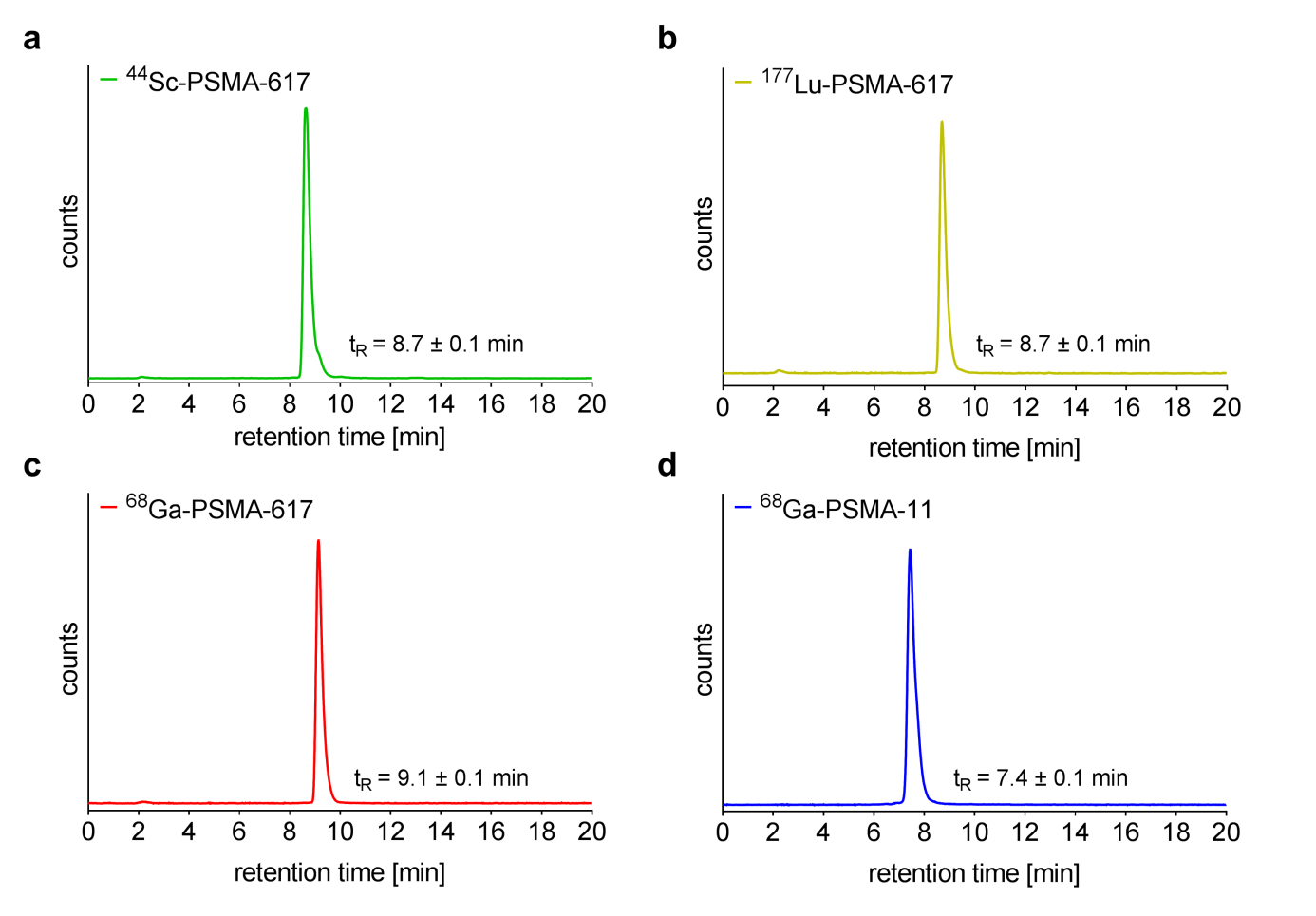


**Fig. S1** Representative chromatograms performed after radiolabeling of the PSMA ligands. The product peaks and their retention times are shown: (**a**) ^44^Sc-PSMA-617, (**b**) ^177^Lu-PSMA-617, (**c**) ^68^Ga-PSMA-617 and (**d**) ^68^Ga-PSMA-11.

**2. Description and culture of PC-3 PIP/flu cells**

Sublines of the androgen-independent PC-3 human prostate cancer xenograft, originally derived from an advanced androgen independent bone metastasis, were used in this study. These sublines had been modified previously to express PSMA at high levels (PSMA^pos^ PC-3 PIP cells), useful to test PSMA-targeted ligands. A mock transfection yielded a PSMA-negative control cell line, which does not express PSMA (PSMA^neg^ PC-3 flu cells) [[1](#_ENREF_1), [2](#_ENREF_2)]. The cells were grown in RPMI cell culture medium supplemented with 10% fetal calf serum, L-glutamine and antibiotics. Additionally, puromycin (2 µg/mL) was added to the cell culture medium to maintain PSMA expression [[3](#_ENREF_3)].

**3. Determination of K_D_ Values using PC-3 PIP Tumor Cells**

The dissociation constants (K_D_ values) were determined in order to assess potential differences in PSMA-binding affinities between the PSMA-617 ligand labeled with ^44^Sc, ^177^Lu and ^68/67^Ga, respectively. Moreover, the PSMA-affinity of ^67/68^Ga-PSMA-11 was also determined for comparison with the ^67/68^Ga-PSMA-617 ligand.

**Experimental Procedure**

Determination of the K_D_ values was performed by saturation binding assays using PSMA^pos^ PC-3 PIP cells. After successful radiolabeling of the PSMA ligands, a 2.5-fold molar excess of the corresponding cold metal was added and the reaction mixture was incubated for another 10 min at 95 °C. Quality control was performed again after reaction of the radioligands with the “cold” metal as described in the main manuscript.

The cells were seeded in 48-well-plates (8 x 10^5^ cells in 500 uL RPMI medium/well) allowing adhesion and growth overnight at 37 °C and 5% CO_2_. For the entire experiment, the well-plates were kept on ice. After removal of the supernatant, the cells were washed once with ice-cold PBS pH 7.4 prior to the addition of different concentrations (1 nM-2 µM) of either ^nat/44^Sc-, ^nat/177^Lu-, ^nat/67/68^Ga-PSMA-617 or ^nat/67/68^Ga-PSMA-11 in ice-cold RPMI medium without supplements. For the determination of non-specific binding, cell samples were incubated with 2-phosphonomethyl pentanedioic acid (2-PMPA, 200 μM). The well-plates were incubated for 30 min at 4 °C. Then, the supernatants were removed and the cells washed twice with ice-cold PBS followed by addition of NaOH (1 M, 600 µL) to each well. The cell suspensions were transferred to 4 mL tubes for measurement in a γ-counter (Wallac Wizard 1480, Perkin Elmer). The K_D_ values were determined by plotting specific-binding (total binding minus unspecific binding) against the molar concentration of the added radioligands followed by nonlinear regression analysis using GraphPad Prism 7 software.

**Results and Conclusions**

K_D_ values of ^nat/44^Sc-PSMA-617, ^nat/67/68^Ga-PSMA-617 and ^nat/67/68^Ga-PSMA-11 were determined using PC-3 PIP tumor cells and compared with the results obtained for ^nat/177^Lu-PSMA-617 (Fig. S2).

The K_D_ values obtained for ^nat/44^Sc-PSMA-617 (33 ± 12 nM) and ^nat/177^Lu-PSMA-617 (39 ± 23 nM) were highly congruent, while ^nat/67/68^Ga-PSMA-617 (72 ± 29) and ^nat/67/68^Ga-PSMA-11 (87 ± 27 nM) showed slightly higher values indicating a somewhat lower affinity.

A number of different protocols for the determination of the PSMA affinity are published in the literature [[4](#_ENREF_4), [5](#_ENREF_5)]. As the outcome of the affinity assays is highly dependent on the experimental set-up, it was decided to present the binding affinities as the inverse molar ratio relative to the value obtained for ^nat/177^Lu-PSMA-617, which was set to 1 as previously suggested by Reddy et al [[6](#_ENREF_6)]. The values for the relative binding affinity are listed and discussed in the main manuscript.


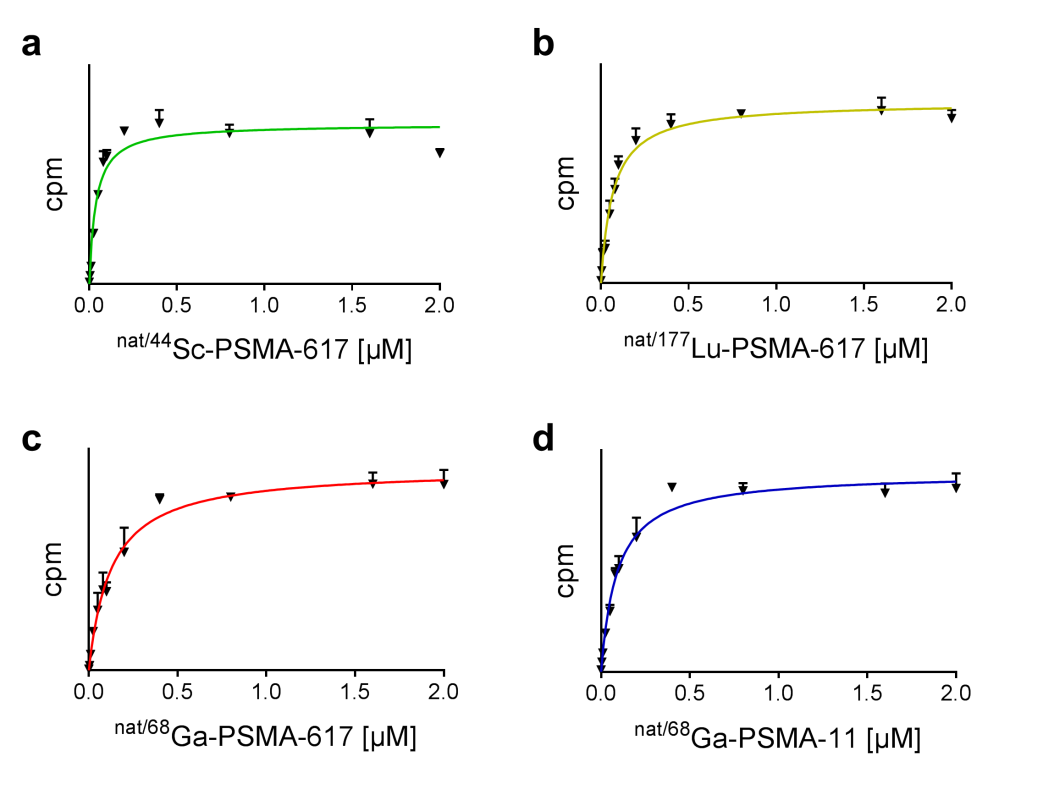


**Fig. S2** Representative graphs of experiments performed to determine K_D_ values showing counts per minute (cpm) measured in cell samples exposed to different concentrations of the respective PSMA radioligand. (**a**, green) ^nat/44^Sc-PSMA-617, (**b**, yellow) ^nat/177^Lu-PSMA-617, (**c**, red) ^nat/68^Ga-PSMA-617 and (**d**, blue) ^nat/68^Ga-PSMA-11.

**4. Biodistribution Studies of ^44^Sc/^177^Lu/^68^Ga-PSMA-617 and ^68^Ga-PSMA-11**

Biodistribution studies of all four radioligands were performed in PC-3 PIP/flu tumor-bearing mice in order to investigate ^44^Sc-PSMA-617 and compare the results with those obtained with ^177^Lu-PSMA-617, ^68^Ga-PSMA-617 and ^68^Ga-PSMA-11. The tissue distribution data are presented in Tables S1-S4 and comparison of the uptake in the tumor and kidneys of all four radioligands is shown in Table S5. The results are discussed in the main manuscript.

**Table S1** Biodistribution of ^44^Sc-PSMA-617 in PC-3 PIP/flu tumor-bearing female nude mice

|  | ^44^Sc-PSMA-617 | | | | |
| --- | --- | --- | --- | --- | --- |
|  | 15 min p.i. | 30 min p.i. | 2 h p.i. | 4 h p.i. | 6 h p.i. |
| Blood | 6.76 ± 0.28 | 2.52 ± 0.43 | 0.21 ± 0.05 | 0.41 ± 0.04 | 0.27 ± 0.02 |
| Heart | 2.79 ± 0.31 | 1.31 ± 0.14 | 0.36 ± 0.04 | 0.53 ± 0.08 | 0.63 ± 0.10 |
| Lung | 5.24 ± 0.30 | 2.06 ± 0.25 | 0.37 ± 0.01 | 0.46 ± 0.01 | 0.51 ± 0.06 |
| Spleen | 3.06 ± 0.18 | 2.20 ± 0.67 | 0.81 ± 0.34 | 0.86 ± 0.22 | 0.90 ± 0.03 |
| Kidneys | 37.7 ± 0.82 | 27.8 ± 3.29 | 5.97 ± 0.90 | 4.14 ± 0.22 | 3.09 ± 0.58 |
| Stomach | 2.25 ± 0.16 | 0.91 ± 0.17 | 0.20 ± 0.05 | 0.41 ± 0.03 | 0.39 ± 0.07 |
| Intestines | 1.89 ± 0.13 | 1.05 ± 0.32 | 0.15 ± 0.04 | 0.18 ± 0.04 | 0.13 ± 0.01 |
| Liver | 1.98 ± 0.19 | 0.80 ± 0.05 | 0.22 ± 0.03 | 0.32 ± 0.04 | 0.31 ± 0.02 |
| Salivary glands | 2.27 ± 0.22 | 1.21 ± 0.14 | 0.36 ± 0.07 | 0.78 ± 0.28 | 0.75 ± 0.17 |
| Muscle | 1.37 ± 0.11 | 1.08 ± 0.20 | 0.36 ± 0.11 | 0.81 ± 0.16 | 0.94 ± 0.08 |
| Bone | 2.75 ± 0.73 | 0.92 ± 0.19 | 0.26 ± 0.13 | 0.69 ± 0.10 | 0.65 ± 0.21 |
| PC-3 PIP Tumor | 36.5 ± 7.44 | 44.4 ± 7.51 | 46.7 ± 4.36 | 51.9 ± 4.05 | 52.0 ± 8.42 |
| PC-3 flu Tumor | 2.14 ± 0.13 | 1.51 ± 0.18 | 0.32 ± 0.16 | 0.40 ± 0.08 | 0.24 ± 0.02 |
| Tumor-to-blood | 5.38 ± 0.90 | 17.6 ± 1.23 | 227 ± 63.4 | 127 ± 21.7 | 194 ± 21.0 |
| Tumor-to-liver | 18.8 ± 5.02 | 55.6 ± 7.36 | 216 ± 33.4 | 166 ± 27.6 | 167 ± 32.5 |
| Tumor-to-kidney | 0.97 ± 0.21 | 1.60 ± 0.22 | 7.98 ± 1.71 | 12.5 ± 1.02 | 17.0 ± 2.58 |

values shown represent the mean ± S.D. of data from three animals (n=3) per cohort

tumor-to-background ratios are calculated for the PSMA-positive PC-3 PIP tumor xenografts

**Table S2** Biodistribution of ^177^Lu-PSMA-617 in PC-3 PIP/flu tumor-bearing female nude mice

|  | ^177^Lu-PSMA-617 | | | | |
| --- | --- | --- | --- | --- | --- |
|  | 15 min p.i. | 30 min p.i. | 2 h p.i. | 4 h p.i. | 6 h p.i. |
| Blood | 7.28 ± 0.32 | 3.05 ± 0.81 | 0.07 ± 0.01 | 0.02 ± 0.00 | 0.02 ± 0.00 |
| Heart | 2.87 ± 0.17 | 1.32 ± 0.28 | 0.06 ± 0.00 | 0.03 ± 0.00 | 0.03 ± 0.00 |
| Lung | 4.76 ± 0.55 | 2.51 ± 0.44 | 0.15 ± 0.03 | 0.07 ± 0.01 | 0.06 ± 0.02 |
| Spleen | 2.85 ± 0.38 | 1.55 ± 0.05 | 0.21 ± 0.03 | 0.15 ± 0.04 | 0.11 ± 0.01 |
| Kidneys | 30.8 ± 4.52 | 22.6 ± 1.78 | 3.97 ± 0.56 | 3.68 ± 1.05 | 2.47 ± 0.28 |
| Stomach | 1.70 ± 0.12 | 0.98 ± 0.35 | 0.08 ± 0.02 | 0.08 ± 0.03 | 0.05 ± 0.01 |
| Intestines | 1.64 ± 0.12 | 1.13 ± 0.45 | 0.14 ± 0.06 | 0.07 ± 0.05 | 0.03 ± 0.00 |
| Liver | 1.63 ± 0.27 | 0.90 ± 0.15 | 0.13 ± 0.02 | 0.09 ± 0.01 | 0.09 ± 0.01 |
| Salivary glands | 2.11 ± 0.19 | 1.01 ± 0.27 | 0.10 ± 0.03 | 0.04 ± 0.01 | 0.04 ± 0.00 |
| Muscle | 1.78 ± 0.21 | 0.75 ± 0.20 | 0.04 ± 0.02 | 0.02 ± 0.00 | 0.02 ± 0.01 |
| Bone | 2.03 ± 0.10 | 1.07 ± 0.27 | 0.12 ± 0.07 | 0.06 ± 0.02 | 0.05 ± 0.01 |
| PC-3 PIP Tumor | 32.3 ± 3.54 | 47.0 ± 3.52 | 45.8 ± 4.02 | 56.0 ± 8.0 | 55.4 ± 1.67 |
| PC-3 flu Tumor | 2.49 ± 0.45 | 1.22 ± 0.32 | 0.13 ± 0.01 | 0.08 ± 0.01 | 0.06 ± 0.01 |
| Tumor-to-blood | 4.45 ± 0.66 | 16.0 ± 3.52 | 633 ± 54.5 | 2315 ± 131 | 2988 ± 351 |
| Tumor-to-liver | 20.4 ± 5.48 | 53.1 ± 6.68 | 346 ± 22.7 | 598 ± 33.2 | 617 ± 66.2 |
| Tumor-to-kidney | 1.06 ± 0.14 | 2.09 ± 0.24 | 11.6 ± 0.87 | 15.7 ± 2.79 | 22.5 ± 1.79 |

values shown represent the mean ± S.D. of data from three animals (n=3) per cohort

tumor-to-background ratios are calculated for the PSMA-positive PC-3 PIP tumor xenografts

**Table S3** Biodistribution of ^68^Ga-PSMA-617 in PC-3 PIP/flu tumor-bearing female nude mice

|  | ^68^Ga-PSMA-617 | | |
| --- | --- | --- | --- |
|  | 15 min p.i. | 30 min p.i. | 2 h p.i. |
| Blood | 6.88 ± 0.89 | 3.46 ± 0.98 | 0.14 ± 0.00 |
| Heart | 2.61 ± 0.49 | 1.47 ± 0.34 | 0.09 ± 0.01 |
| Lung | 4.70 ± 0.09 | 2.55 ± 0.51 | 0.20 ± 0.00 |
| Spleen | 3.92 ± 0.28 | 2.50 ± 0.82 | 0.91 ± 0.14 |
| Kidneys | 22.8 ± 0.81 | 15.5 ± 3.46 | 3.48 ± 0.18 |
| Stomach | 1.48 ± 0.09 | 1.09 ± 0.40 | 0.16 ± 0.11 |
| Intestines | 1.55 ± 0.19 | 0.98 ± 0.28 | 0.12 ± 0.03 |
| Liver | 4.74 ± 0.43 | 2.91 ± 0.65 | 1.60 ± 0.05 |
| Salivary glands | 2.30 ± 0.37 | 1.06 ± 0.23 | 0.14 ± 0.03 |
| Muscle | 1.53 ± 0.24 | 0.80 ± 0.10 | 0.05 ± 0.01 |
| Bone | 1.72 ± 0.06 | 1.00 ± 0.17 | 0.09 ± 0.02 |
| PC-3 PIP Tumor | 28.7 ± 5.26 | 48.5 ± 8.51 | 55.8 ± 14.2 |
| PC-3 flu Tumor | 2.30 ± 0.18 | 1.23 ± 0.35 | 0.18 ± 0.03 |
| Tumor-to-blood | 4.15 ± 0.24 | 16.9 ± 4.22 | 387 ± 98.1 |
| Tumor-to-liver | 6.02 ± 0.59 | 19.0 ± 2.32 | 35.2 ± 9.94 |
| Tumor-to-kidney | 1.26 ± 0.25 | 3.62 ± 0.81 | 15.9 ± 3.26 |

values shown represent the mean ± S.D. of data from three animals (n=3) per cohort

tumor-to-background ratios are calculated for the PSMA-positive PC-3 PIP tumor xenografts

**Table S4** Biodistribution of ^68^Ga-PSMA-11 in PC-3 PIP/flu tumor-bearing female nude mice

|  | ^68^Ga-PSMA-11 | | |
| --- | --- | --- | --- |
|  | 15 min p.i. | 30 min p.i. | 2 h p.i. |
| Blood | 5.05 ± 0.58 | 2.55 ± 0.72 | 0.08 ± 0.01 |
| Heart | 2.45 ± 0.29 | 1.31 ± 0.19 | 0.18 ± 0.03 |
| Lung | 3.87 ± 0.22 | 2.23 ± 0.33 | 0.31 ± 0.10 |
| Spleen | 5.33 ± 1.78 | 4.76 ± 1.13 | 2.18 ± 0.38 |
| Kidneys | 74.7 ± 7.02 | 86.7 ± 14.6 | 58.8 ± 7.62 |
| Stomach | 1.42 ± 0.15 | 0.85 ± 0.19 | 0.35 ± 0.17 |
| Intestines | 1.42 ± 0.07 | 1.20 ± 0.24 | 0.95 ± 0.86 |
| Liver | 2.00 ± 0.32 | 1.55 ± 0.04 | 0.56 ± 0.04 |
| Salivary glands | 2.73 ± 0.27 | 1.59 ± 0.18 | 0.39 ± 0.01 |
| Muscle | 1.21 ± 0.11 | 0.67 ± 0.06 | 0.06 ± 0.01 |
| Bone | 1.85 ± 0.20 | 0.81 ± 0.14 | 0.06 ± 0.00 |
| PC-3 PIP Tumor | 31.5 ± 13.0 | 49.3 ± 4.97 | 40.0 ± 2.55 |
| PC-3 flu Tumor | 2.19 ± 0.05 | 1.04 ± 0.17 | 0.13 ± 0.02 |
| Tumor-to-blood | 6.10 ± 1.84 | 20.5 ± 6.40 | 536 ± 43.7 |
| Tumor-to-liver | 15.3 ± 3.96 | 31.7 ± 2.48 | 71.3 ± 7.48 |
| Tumor-to-kidney | 0.41 ± 0.14 | 0.58 ± 0.10 | 0.69 ± 0.12 |

values shown represent the mean ± S.D. of data from three animals (n=3) per cohort

tumor-to-background ratios are calculated for the PSMA-positive PC-3 PIP tumor xenografts

**Table S5** Tumor and kidney uptake of the PSMA-targeted radioligands

|  | 15 min after injection | | | |
| --- | --- | --- | --- | --- |
| PSMA ligand | ^44^Sc-PSMA-617 | ^177^Lu-PSMA-617 | ^68^Ga-PSMA-617 | ^68^Ga-PSMA-11 |
| PC-3 PIP | 36.5 ± 7.44 | 32.3 ± 3.54 | 28.7 ± 5.26 | 31.50 ± 13.0 |
| PC-3 flu | 2.14 ± 0.13 | 2.49 ± 0.45 | 2.30 ± 0.18 | 2.19 ± 0.05 |
| Kidney | 37.7 ± 0.82 | 30.8 ± 4.52 | 22.8 ± 0.81 | 74.7 ± 7.02 |
|  | 30 min after injection | | | |
| PSMA ligand | ^44^Sc-PSMA-617 | ^177^Lu-PSMA-617 | ^68^Ga-PSMA-617 | ^68^Ga-PSMA-11 |
| PC-3 PIP | 44.4 ± 7.51 | 47.0 ± 3.52 | 48.5 ± 8.51 | 49.3 ± 4.97 |
| PC-3 flu | 1.51 ± 0.18 | 1.22 ± 0.32 | 1.23 ± 0.35 | 1.04 ± 0.17 |
| Kidney | 27.8 ± 3.29 | 22.6 ± 1.78 | 15.5 ± 3.46 | 86.7 ± 14.6 |
|  | 2 h after injection | | | |
|  | ^44^Sc-PSMA-617 | ^177^Lu-PSMA-617 | ^68^Ga-PSMA-617 | ^68^Ga-PSMA-11 |
| PC-3 PIP | 46.7 ± 4.36 | 45.8 ± 4.02 | 55.8 ± 14.2 | 40.0 ± 2.55 |
| PC-3 flu | 0.32 ± 0.16 | 0.13 ± 0.01 | 0.18 ± 0.03 | 0.13 ± 0.02 |
| Kidney | 5.97 ± 0.90 | 3.97 ± 0.56 | 3.48 ± 0.18 | 58.8 ± 7.62 |

**5. Imaging Studies in PC-3 PIP/flu Tumor-Bearing Mice**

PET/CT (^44^Sc and ^68^Ga) and SPECT/CT (^177^Lu) studies were performed in order to compare the tissue distribution 2 hours after injection of the PSMA-targeted radioligands in the same tumor-mouse model.

**Experimental procedure**

PET/CT scans were performed with a small-animal bench-top PET/CT scanner (G8, Sofie Biosciences, Culver City, California, U.S. and Perkin Elmer, Massachusetts, U.S.). The energy window ranged from 150 keV to 650 keV. Mice were injected intravenously with a physiological saline solution (100-200 μL) containing the ^44^Sc- and ^68^Ga-labeled PSMA ligands. Static whole-body PET scans were performed at 30 min, 2 h, 4 h and 6 h after injection of ^44^Sc-PSMA-617 (~5 MBq, 1 nmol or 3 nmol) and 30 min and 2 h after injection of ^68^Ga-PSMA-617 (~5 MBq, 1 nmol) and ^68^Ga-PSMA-11 (~5 MBq, 1 nmol), respectively. The PET scans lasted for 10 min (^68^Ga-PSMA-617/11) and 20 min (^44^Sc-PSMA-617), respectively, followed by a CT scan of 1.5 min. The data were corrected for random coincidences, decay and dead time. The images were acquired using G8 acquisition software (version 2.0.0.10) and reconstructed with maximum-likelihood expectation maximization (MLEM).

SPECT/CT imaging studies were performed using a small-animal SPECT/CT camera (NanoSPECT/CT^TM^, Mediso Medical Imaging Systems, Budapest, Hungary). A SPECT scan of 45 min duration was performed 2 h after injection of ^177^Lu-PSMA-617 (~50 MBq, 1 nmol) followed by a CT scan of 7.5 min. The image was acquired using Nucline Software (version 1.02, Bioscan Inc., Poway, California, US) and the reconstruction was performed using HiSPECT software (version 1.4.3049, Scivis GmbH, Göttingen, Germany) using the x-ray and γ-energies of 56.1 keV ± 10%, 112.9 keV ± 10% and 208.4 keV ± 10% keV for ^177^Lu.

During the scans the mice were anesthetized using a mixture of isoflurane and oxygen. All images were prepared using *VivoQuant* post-processing software (version 2.10, inviCRO Imaging Services and Software, Boston U.S.). A Gauss post-reconstruction filter (full width at half maximum = 1 mm) was applied to the PET and SPECT images. The images are presented with the scale adjusted to allow visualization of the most important organs and tissues, usually by cutting 0.5-1% of the lower scale.

**Results and conclusions**

The tissue distribution of ^44^Sc-PSMA-617, ^177^Lu-PSMA-617 and ^68^Ga-PSMA-617 was largely the same with significant accumulation of the radioligands in the tumor xenograft of the right shoulder (PSMA^pos^ PC-3 PIP tumor xenograft) but no uptake in the PSMA^neg^ PC-3 flu tumor xenograft on the left shoulder (Fig. S3). Retention of radioactivity in the kidneys was largely absent or only shown when the scale was adjusted. The tissue distribution of ^68^Ga-PSMA-11 was clearly different showing high accumulation of radioactivity in the renal tissue as it has been previously demonstrated by other groups [[7](#_ENREF_7), [8](#_ENREF_8)] as well as in our post-mortem biodistribution studies (Table S4).


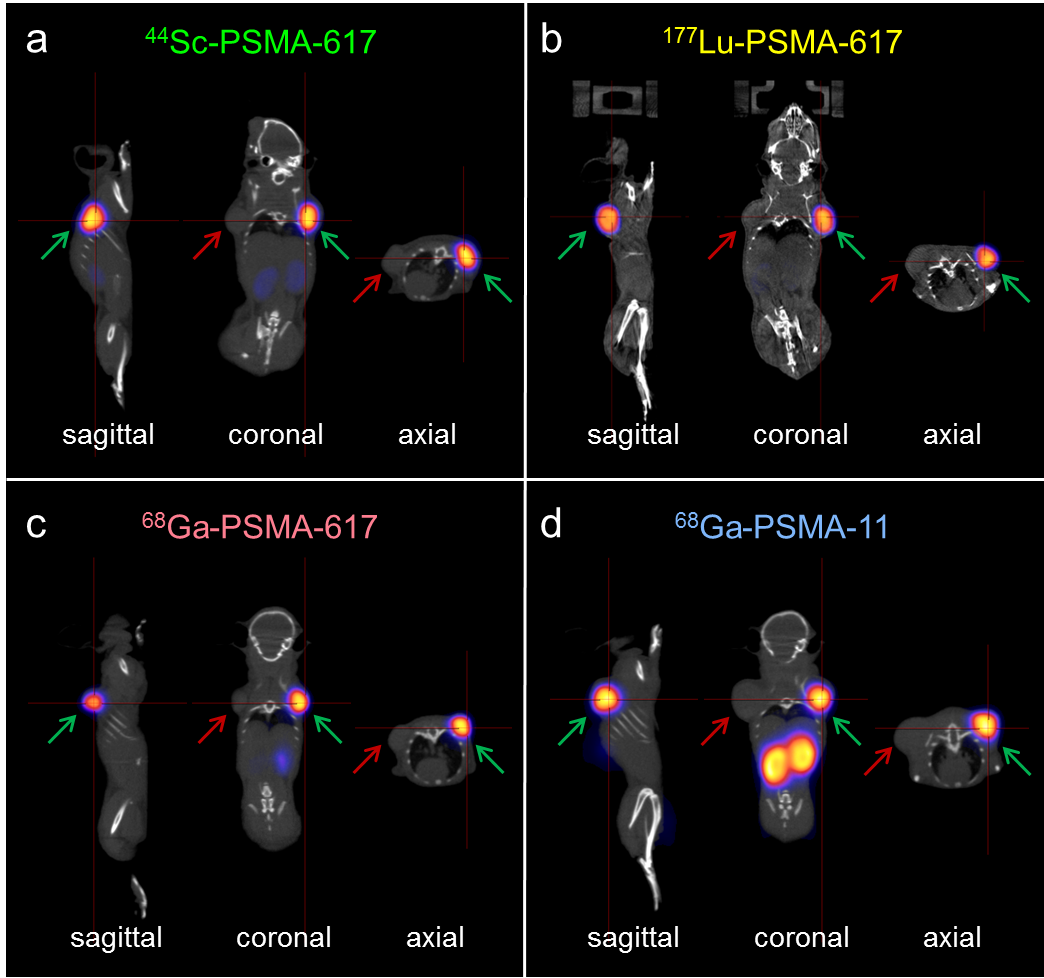


**Fig. S3** Images (PET/CT and SPECT/CT, respectively) as sagittal, coronal and axial sections of PC-3 PIP/flu tumor-bearing mice 2 h after injection of (**a**) ^44^Sc-PSMA-617, (**b**) ^177^Lu-PSMA-617, (**c**) ^68^Ga-PSMA-617 and (**d**) ^68^Ga-PSMA-11. Each mouse has a PSMA^pos^ tumor xenograft (PC-3 PIP – green arrow; crosshair place in this tumor) on the right shoulder and a PSMA^neg^ tumor xenograft (PC-3 flu, red arrow) on the left shoulder.

**References**

1. Wu P, Kudrolli TA, Chowdhury WH, Liu MM, Rodriguez R, Lupold SE. Adenovirus targeting to prostate-specific membrane antigen through virus-displayed, semirandom peptide library screening. Cancer Res. 2010;70(23):9549-53. doi:10.1158/0008-5472.CAN-10-1760.

2. Chang S, Reuter VE, Heston WDW, Bander NH, Grauer LS, Gaudin PB. Five different anti-prostate-specific membrane antigen (PSMA) antibodies confirm PSMA expression in tumor-associated neovasculature. Cancer Res. 1999:3192–8.

3. Banerjee SR, Pullambhatla M, Foss CA, Nimmagadda S, Ferdani R, Anderson CJ et al. ^64^Cu-labeled inhibitors of prostate-specific membrane antigen for PET imaging of prostate cancer. J Med Chem. 2014;57(6):2657-69. doi:10.1021/jm401921j.

4. Kularatne SA, Wang K, Santhapuram HK, Low PS. Prostate-specific membrane antigen targeted imaging and therapy of prostate cancer using a PSMA inhibitor as a homing ligand. Mol Pharm. 2009;6(3):780-9. doi:10.1021/mp900069d.

5. Benesova M, Schäfer M, Bauder-Wust U, Afshar-Oromieh A, Kratochwil C, Mier W et al. Preclinical evaluation of a tailor-made DOTA-conjugated PSMA inhibitor with optimized linker moiety for imaging and endoradiotherapy of prostate cancer. J Nucl Med. 2015;56(6):914-20. doi:10.2967/jnumed.114.147413.

6. Reddy JA, Xu LC, Parker N, Vetzel M, Leamon CP. Preclinical evaluation of ^99m^Tc-EC20 for imaging folate receptor-positive tumors. J Nucl Med. 2004;45(5):857-66.

7. Eder M, Schäfer M, Bauder-Wust U, Hull WE, Wängler C, Mier W et al. ^68^Ga-complex lipophilicity and the targeting property of a urea-based PSMA inhibitor for PET imaging. Bioconjug Chem. 2012;23(4):688-97. doi:10.1021/bc200279b.

8. Afshar-Oromieh A, Hetzheim H, Kübler W, Kratochwil C, Giesel FL, Hope TA et al. Radiation dosimetry of ^68^Ga-PSMA-11 (HBED-CC) and preliminary evaluation of optimal imaging timing. Eur J Nucl Med Mol Imaging. 2016;43(9):1611-20. doi:10.1007/s00259-016-3419-0.
